# Highly Rearranged Karyotypes and Multiple Sex Chromosome Systems in Armored Catfishes from the Genus *Harttia* (Teleostei, Siluriformes)

**DOI:** 10.3390/genes11111366

**Published:** 2020-11-18

**Authors:** Geize Aparecida Deon, Larissa Glugoski, Marcelo Ricardo Vicari, Viviane Nogaroto, Francisco de Menezes Cavalcante Sassi, Marcelo de Bello Cioffi, Thomas Liehr, Luiz Antonio Carlos Bertollo, Orlando Moreira-Filho

**Affiliations:** 1 Laboratório de Citogenética de Peixes, Departamento de Genética e Evolução, Universidade Federal de São Carlos, São Carlos SP 13565-905, Brazil; geizedeon@hotmail.com (G.A.D.); lariglugoski@hotmail.com (L.G.); fmcsassi@estudante.ufscar.br (F.d.M.C.S.); mbcioffi@ufscar.br (M.d.B.C.); bertollo@ufscar.br (L.A.C.B.); omfilho@ufscar.br (O.M.-F.); 2 Laboratório de Biologia Cromossômica, Estrutura e Função, Departamento de Biologia Estrutural, Molecular e Genética, Universidade Estadual de Ponta Grossa, Ponta Grossa PR 84030-900, Brazil; vicarimr@pq.cnpq.br (M.R.V.); vivianenogaroto@hotmail.com (V.N.); 3 Institute of Human Genetics, University Hospital Jena, 07747 Jena, Germany

**Keywords:** chromosomal rearrangements, comparative genomic hybridization, fish mapping, fish species, karyotype evolution, sex chromosomes

## Abstract

*Harttia* comprises an armored catfish genus endemic to the Neotropical region, including 27 valid species with low dispersion rates that are restricted to small distribution areas. Cytogenetics data point to a wide chromosomal diversity in this genus due to changes that occurred in isolated populations, with chromosomal fusions and fissions explaining the 2n number variation. In addition, different multiple sex chromosome systems and rDNA loci location are also found in some species. However, several *Harttia* species and populations remain to be investigated. In this study, *Harttia intermontana* and two still undescribed species, morphologically identified as *Harttia* sp. 1 and *Harttia* sp. 2, were cytogenetically analyzed. *Harttia intermontana* has 2n = 52 and 2n = 53 chromosomes, while *Harttia* sp. 1 has 2n = 56 and 2n = 57 chromosomes in females and males, respectively, thus highlighting the occurrence of an XX/XY_1_Y_2_ multiple sex chromosome system in both species. *Harttia* sp. 2 presents 2n = 62 chromosomes for both females and males, with fission events explaining its karyotype diversification. Chromosomal locations of the rDNA sites were also quite different among species, reinforcing that extensive rearrangements had occurred in their karyotype evolution. Comparative genomic hybridization (CGH) experiments among some *Harttia* species evidenced a shared content of the XY_1_Y_2_ sex chromosomes in three of them, thus pointing towards their common origin. Therefore, the comparative analysis among all *Harttia* species cytogenetically studied thus far allowed us to provide an evolutionary scenario related to the speciation process of this fish group.

## 1. Introduction

Fishes exhibit the greatest biodiversity among the vertebrates, constituting a useful model for studying several evolutionary questions [1]. Particularly, the large river networks found in the Neotropical region contain the world’s richest biodiversity. Despite the large geographic distribution of the Neotropical fish families, different species are found inhabiting adjacent river basins split by vicariant events millions of years ago [2]. In the same way, species inhabiting small streams, with limited migration opportunities, tend to present an increased rate of speciation [3], even in parapatric populations [4].

One of these examples relies on the genus *Harttia* (Siluriformes, Loricariidae, Loricariinae), an endemic and widespread group throughout many South American river basins [5]. These species have a sedentary lifestyle and reduced vagility, leading them to inhabit specific sections of the river and to form small local populations [6].

Studied species indicate a wide variation on the diploid number (2n) in *Harttia*, ranging from 2n = 52 to 62 chromosomes, with the occurrence of interstitial telomeric sites (ITS) as vestiges of chromosomal changes, different ribosomal genes distributions on the karyotypes, occurrence of B chromosomes, and multiple sex chromosome systems (Table 1). Based on the molecular phylogenetic inferences in the Harttiini tribe [7], a scenario for *Harttia* karyotype diversification was proposed by Blanco et al. [8]. In this scenario, a putative ancestral karyotype would have 2n = 58 chromosomes and no heteromorphic sex chromosomes, such as that found in *Harttia kronei* [8]. From such a karyotype, reductions in 2n number by chromosome fusions were proposed in the diversification of lineages, until the lowest number of chromosomes, 2n = 52, found in *H. carvalhoi* females was reached [8].

Furthermore, the chromosomal rearrangements in *Harttia* species culminated in different kinds of sex chromosome systems: (*i*) an XX/XY_1_Y_2_ system in *H. carvalhoi* [9]; (*ii*) an X_1_X_1_X_2_X_2_/X_1_X_2_Y system in *Harttia punctata*, *Harttia duriventris*, and *Harttia villasboas* [10,11]; and (*iii*) a neo XX/XY system in *Harttia rondoni* [11]. In accordance with the two branches of *Harttia* species [7], these sex chromosome systems followed independent evolutionary origins [8,11]. While *H. carvalhoi* is grouped with *H. kronei*, *H. longipinna*, *H. loricariformis***,** and some other species distributed in southern and southeastern Brazil, *H. punctata*, *H. rondoni*, *H. duriventris*, and *H. villasboas* are grouped in a different branch with other species from the north and northeast Brazilian drainages [7].

Simple sex chromosome systems are proposed to have originated by an inversion event or by the accumulation of transposable elements in one homologue of a proto sex chromosome pair, thus promoting a cross-over restricted region able to differentiate in a sex-specific chromosomal segment [12,13,14]. Additionally, with regard to multiple sex chromosomes, different types of chromosomal rearrangements (such as centric fusions, centric fissions, pericentric inversions, translocations, tandem translocations), usually associated with autosomes and proto-sex chromosomes, have already been proposed to explain the origin of different types of systems [9,15,16,17,18,19]. More recently, and with the aim of discovering the evolutionary origin of the sex chromosomes systems, molecular cytogenetics approaches such as whole chromosome painting (WCP) and comparative genome hybridization (CGH) have been successfully used in some Neotropical fish species [19,20,21,22].

In this study, we provide a set of conventional and molecular cytogenetic approaches (Giemsa staining, C-banding, repetitive DNA mapping by FISH, comparative genomic hybridization (CGH)), in an attempt to advance the knowledge of the processes that have shaped the chromosomal evolution in the genus *Harttia*. Data allowed for a comprehensive perspective of the chromosomal diversity and evolutionary trends inside this group, in addition to a description of two other new rare occurrences of the XX/XY_1_Y_2_ sex chromosome system among fishes.

## 2. Materials and Methods

### 2.1. Specimens

**Figure 1:**
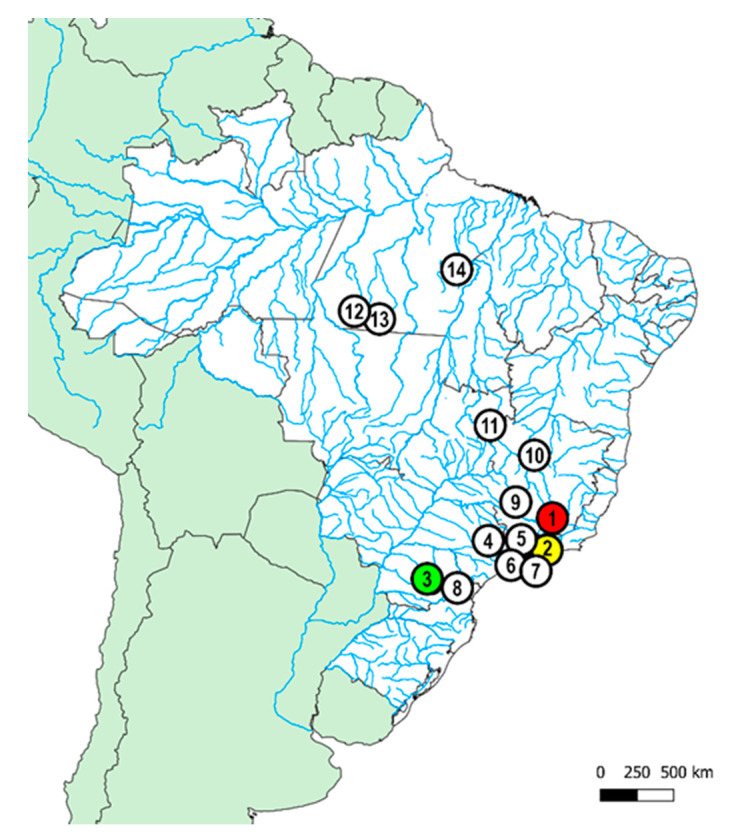
Partial map of South America highlighting the Brazilian collection sites of the three *Harttia* species analyzed in the present work (color circles) named: 1. *H. intermontana* (red circle); 2. *Harttia* sp. 1 (yellow circle); and 3. *Harttia* sp. 2 (green circle). The white circles represent other *Harttia* species previously studied: 4. *H. absaberi*, 5. *H. carvalhoi*, 6. *H. gracilis*, 7. *H. loricariformis*, 8. *H. kronei*, 9. *H. torrenticola*, 10. *H. longipinna*, 11. *H. punctata*; 12. *H. rondoni*; 13. *H. villasboas,* and 14. *H. duriventris*. Map created using QGis 3.4.3 and Adobe Photoshop CC 2020.

Three *Harttia* species not yet studied were investigated. Their collection sites, number, and sex of individuals are presented in Figure 1 and Table 2. Figure 1 also depicts the Brazilian distribution of other *Harttia* species previously studied. Fishes were collected with the authorization of the Chico Mendes Institute for Biodiversity Conservation (ICMBIO), System of Authorization and Information about Biodiversity (SISBIO-License Ns^o^. 10538-3 and 15117-1), and National System of Genetic Resource Management and Associated Traditional Knowledge (SISGEN-A96FF09). The species were properly identified by Dr. Oswaldo Oyakawa (curator of the fish collection of the Museu de Zoologia da Universidade de São Paulo (MZUSP), with expertise on *Harttia* taxonomy. One of the three species corresponds to *Harttia intermontana*, and the other two correspond to new species that have not yet been described, here named as *Harttia* sp. 1 and *Harttia* sp. 2.

### 2.2. Chromosome Preparations and C-Banding

Mitotic chromosomes were obtained from cells of the anterior region of the kidney after in vivo colchicine treatment according to the protocol described in Bertollo et al. [26]. The experiments followed ethical and anesthesia procedures that were approved by the Ethics Committee on Animal Experimentation of the Universidade Federal de São Carlos (Process number CEUA 1853260315). The C-positive heterochromatin (C-banding) was identified according to Sumner [27] with some modifications according to Lui et al. [28].

### 2.3. Fluorescence In Situ Hybridization (FISH)

Two tandemly arrayed rDNA probes were obtained by PCR from the nuclear DNA of *Harttia intermontana*. The 5S rDNA probe included 120 base pairs (bp) of the 5S rRNA transcript region and 200 bp of a non-transcribed spacer (NTS), isolated according to Pendás et al. [29]. The 18S rDNA probe contained a 1400 bp segment of the 18S rRNA gene and was isolated following Cioffi et al. [30]. The probes were directly labeled with the Nick-Translation mix kit (Jena Bioscience, Jena, Germany) using ATTO550-dUTP for the 5S rDNA and AF488-dUTP for the 18S rDNA, according to the manufacturer’s manual. Telomeric (TTAGGG)n sequences were also mapped using the DAKO Telomere PNA FISH Kit/FITC (DAKO, Glostrup, Denmark). FISH experiments followed the methodology described in Yano et al. [31].

### 2.4. Comparative Genomic Hybridization (CGH)

The total genomic DNA (gDNA) from male and female specimens of *H. intermontana*, *Harttia* sp. 1, and *H. carvalhoi* were extracted from liver tissues by the standard phenol-chloroform-isoamyl alcohol method [32]. The CGH experiments were focused on inter and intraspecific comparisons, with special emphasis on the XY_1_Y_2_ sex chromosomes. In the first set of experiments (intraspecific genomic comparisons), the male-derived gDNA of *H. intermontana* and *Harttia* sp. 1was labeled by nick translation (Jena Bioscience) with ATTO550-dUTP, while female gDNA was labeled with Atto488-dUTP. Repetitive sequences were blocked in all experiments by using unlabeled C_0_t-1 DNA (i.e., a fraction of genomic DNA enriched for highly and moderately repetitive sequences), prepared according to Zwick et al. [33]. The final hybridization mixture was applied on each slide, which was composed of male- and female-derived gDNAs (500 ng each), plus 25 μg of female-derived C_0_t-1 DNA from the respective species. The probe was ethanol-precipitated, and the dry pellets were resuspended in a hybridization buffer containing 50% formamide, 2× SSC, 10% SDS, 10% dextran sulfate, and Denhardt’s buffer, pH 7.0. In the second set of experiments (interspecific genomic comparisons), the gDNA samples of all-male specimens now analyzed (plus the gDNA of *H. carvalhoi*, another species harboring the same multiple XY_1_Y_2_ sex system) were hybridized against metaphase chromosomes of *H. intermontana*. For this purpose, male-derived gDNA of *H. intermontana* was labeled with Atto550-dUTP by nick translation (Jena Bioscience), while the gDNA samples of the other two species were labeled with Atto488-dUTP (*Harttia* sp. 1) and Atto425-dUTP (*H. carvalhoi*) also by nick translation (Jena Bioscience). The three probes were hybridized simultaneously, and the final probe cocktail was composed of 500 ng of the male-derived gDNA of each *H. intermontana, Harttia* sp. 1, and *H. carvalhoi* species and 10 μg of the female-derived C_0_t-1 DNA of each species. The chosen ratio of probe vs. C_0_t-1 DNA amount was based on fish experiments previously performed in our laboratory [19,34,35,36]. The CGH experiments followed the methodology described in Symonová et al. [37].

### 2.5. Microscopic Analyses and Image Processing

At least 30 metaphase spreads per individual was analyzed to confirm the 2n, karyotype structure, and CGH results. Images were captured using an Olympus BX50 light microscope (Olympus Corporation, Ishikawa, Japan), with CoolSNAP camera, and the images were processed using the Image-Pro Plus 4.1 software (Media Cybernetics, Silver Spring, MD, USA). Chromosomes were classified as metacentric (m); submetacentric (sm); subtelocentric (st), or acrocentric (a) according to Levan et al. [38] and arranged according to decreasing size in the karyotypes. The fundamental number (FN), or number of chromosome arms, was achieved considering just acrocentrics as having a single chromosome arm.

## 3. Results

### 3.1. Karyotypes, C-Banding, and Sex Chromosomes

**Figure 2:**
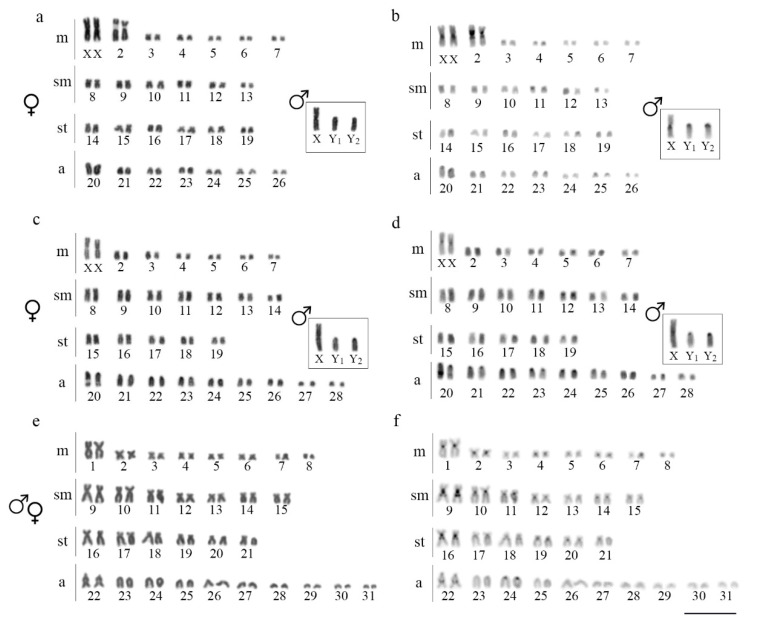
Karyotypes of *H. intermontana* (**a**,**b**), *Harttia* sp. 1 (**c**,**d**), and *Harttia* sp. 2 (**e**,**f**), showing sequentially Giemsa-stained (**a**,**c**,**e**) and C-banded (**b**,**d**,**f**) chromosomes. Insets depict the male sex chromosomes. Bar = 5 μm.

All *H. intermontana* females have 2n = 52 chromosomes (14m + 12sm + 12st + 14a; NF = 90) and all males have 2n = 53 chromosomes (13m + 12sm + 13st + 15a, NF = 91). Similarly, *Harttia* sp. 1 also differs in female and male karyotypes, with 2n = 56 (14m + 14sm + 10st + 18a; NF = 94) and 2n = 57 (13m + 14sm + 10st + 20a; NF = 94), respectively. In both cases, the sex-specific karyotypes are due the occurrence of an XX/XY_1_Y_2_ multiple sex chromosome system, where the X chromosome corresponds to a large metacentric, and the Y_1_ to a medium-size acrocentric. In its turn, the Y_2_ chromosome corresponds to a medium-size subtelocentric in *H. intermontana* and to an acrocentric chromosome in *Harttia* sp. 1 (Figure 2a,c). Additionally, *Harttia* sp. 2 has 2n = 62 chromosomes (16m + 14sm + 12st + 20a; NF = 104) in both sexes, without morphologically differentiated sex chromosomes (Figure 2e).

A small amount of C-positive heterochromatin was found in the three species, mostly in the centromeric/pericentromeric regions of some chromosome pairs (Figure 2b,d,f), without specific accumulation in the sex chromosomes of *H. intermontana* and *Harttia* sp. 1 (Figure 2b,d).

### 3.2. Chromosomal Distribution of rDNAs and Telomeric Repeats

**Figure 3:**
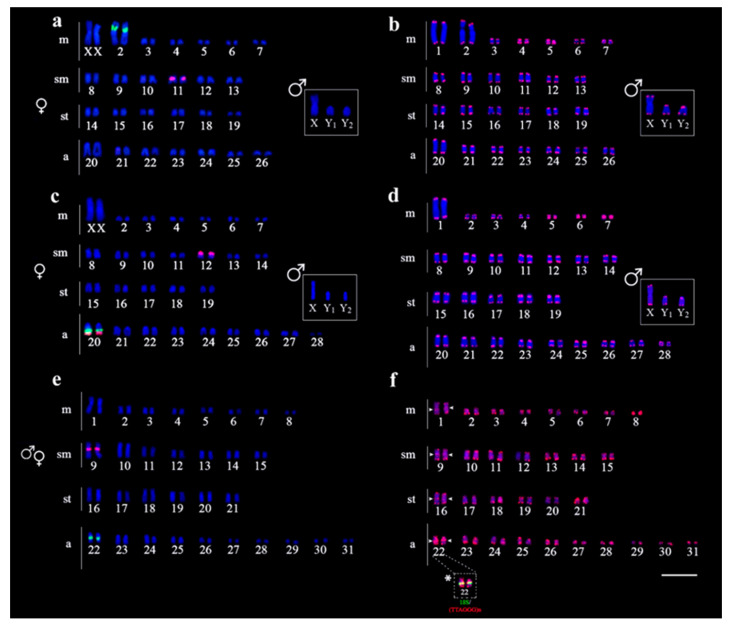
Karyotypes of *H. intermontana* (**a**,**b**), *Harttia* sp. 1 (**c**,**d**), and *Harttia* sp. 2 (**e**,**f**) arranged after FISH with 5S rDNA (red) and 18S rDNA (green) probes (**a**,**c**,**e**), and telomeric (TTAGGG)n probe (**b**,**d**,**f**). Inserts depict the male sex chromosomes. Arrowheads indicate the interstitial telomeric sites (ITS) locations and the (*) signals the joint localization of the 18S and ITS sites. Bar = 5 μm.

Differentiation in number and location of the 5S and 18S rDNA sequences was found among the three species. In *H. intermontana* and *Harttia* sp. 2, a single locus of 5S rDNA occurs, but in different chromosomes, i.e., in the submetacentric pairs 11 and 9, respectively. In *Harttia* sp. 1, there are two 5S rDNA loci, one of which is located in the submetacentric pair 12, and the other in the acrocentric pair 20, with a syntenic location with the 18S rDNA in the latter (Figure 3).

The 18S rDNA probe was detected in a single locus in all species, but was also found in different chromosomal locations as follows: in the short arms of the second metacentric pair in *H. intermontana*; in the long arms of the acrocentric pair 20 in *Harttia* sp. 1, and in the long arms of the acrocentric pair 22 in *Harttia* sp. 2. No differences in the number and site positions of rDNA were detected between males and females (Figure 3).

Hybridization with the (TTAGGG)n probe evidenced signals only in the telomeric regions of all chromosomes, without ITS in *H. intermontana* and *Harttia* sp. 1 (Figure 3b,d). However, in *Harttia* sp. 2, four ITS were located in the long arms of the chromosome pairs 1, 9, 16, and 22. A double-FISH using both telomeric and 18S rDNA probes revealed that these sequences present a syntenic location in the chromosome pair 22 (Figure 3f).

### 3.3. Intraspecific and Interspecific Comparative Genomic Hybridizations

**Figure 4:**
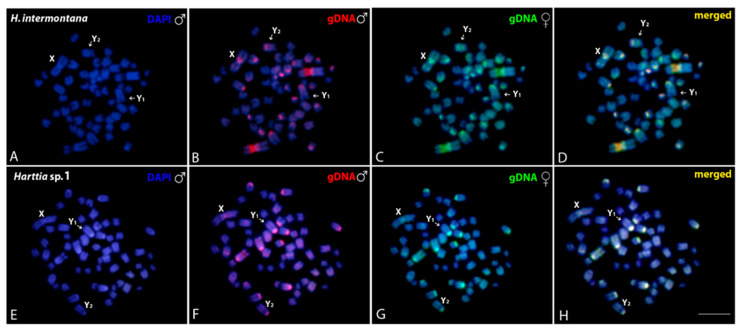
Mitotic chromosome spreads of males *H. intermontana* (**A**–**D**) and *Harttia* sp. 1 (**E**–**H**) after intraspecific genomic hybridizations, with male- and female-derived genomic probes hybridized together for each species. The first column (**A**,**E**): DAPI images (blue); Second column (**B**,**F**): hybridization pattern for the male-derived probe (red); Third column (**C**,**G**): hybridization pattern for the female-derived probe (green); Fourth column (**D**,**H**): merged images for both genomic probes and DAPI staining. The common genomic regions for males and females are depicted in yellow. Arrows indicate the sex chromosomes. Bar = 10 μm.

**Figure 5:**
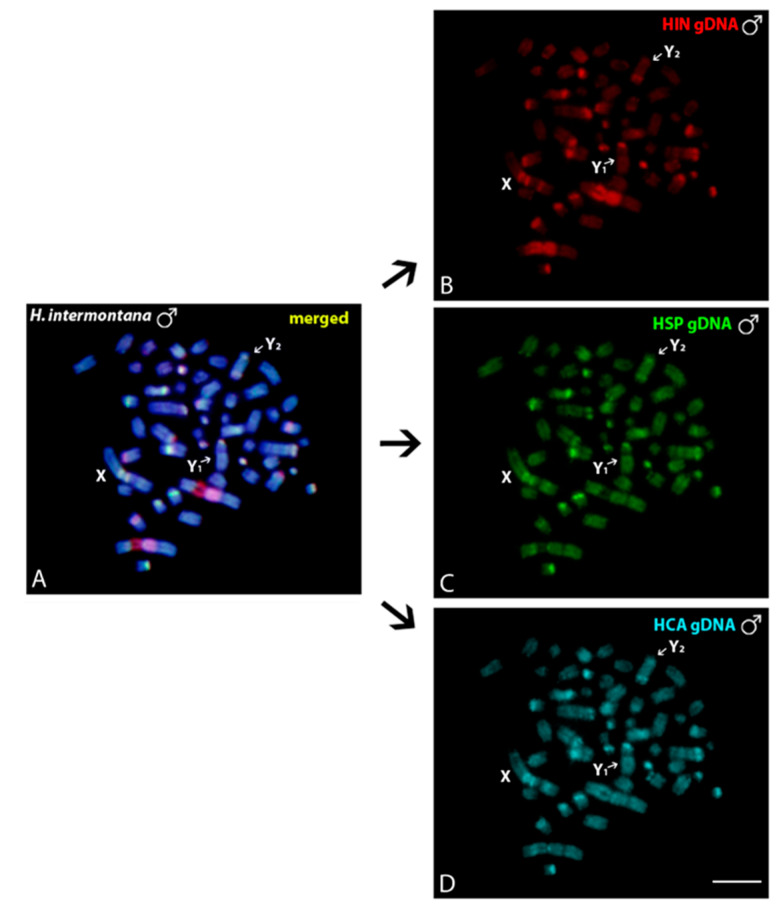
Mitotic chromosome spreads of males from *H. intermontana* (**A**–**D**) after comparative genomic hybridization (CGH): interspecific hybridizations probed with a male-derived genomic probe from *H. intermontana* (**B**), *Harttia* sp. 1 (**C**), and *H. carvalhoi* (**D**). (**A**) depicts the merged images of the genomic probes and DAPI staining. The common genomic regions for male and female are depicted in yellow. Sex chromosomes are indicated. Bar = 10 μm.

Intraspecific genomic comparisons between males (Figure 4b,f) and females (Figure 4c,g) of *H. intermontana* and *Harttia* sp. 1 showed an overlapped hybridization, mainly in the centromeric and pericentromeric regions of almost all chromosomes (Figure 4d,h). A strong binding preference for the 18S rDNA cluster occurs in *H. intermontana* (Figure 4b,c) and no sex-specific region was evidenced in both experiments. Interspecific comparisons of the gDNA of *H. intermontana*, *H. carvalhoi*, and *Harttia* sp. 1, all of them bearing an XY_1_Y_2_ sex system, did not detect species-specific regions in the sex chromosomes (Figure 5).

## 4. Discussion

### 4.1. Numerical Chromosome Changes in Harttia Species

**Figure 6:**
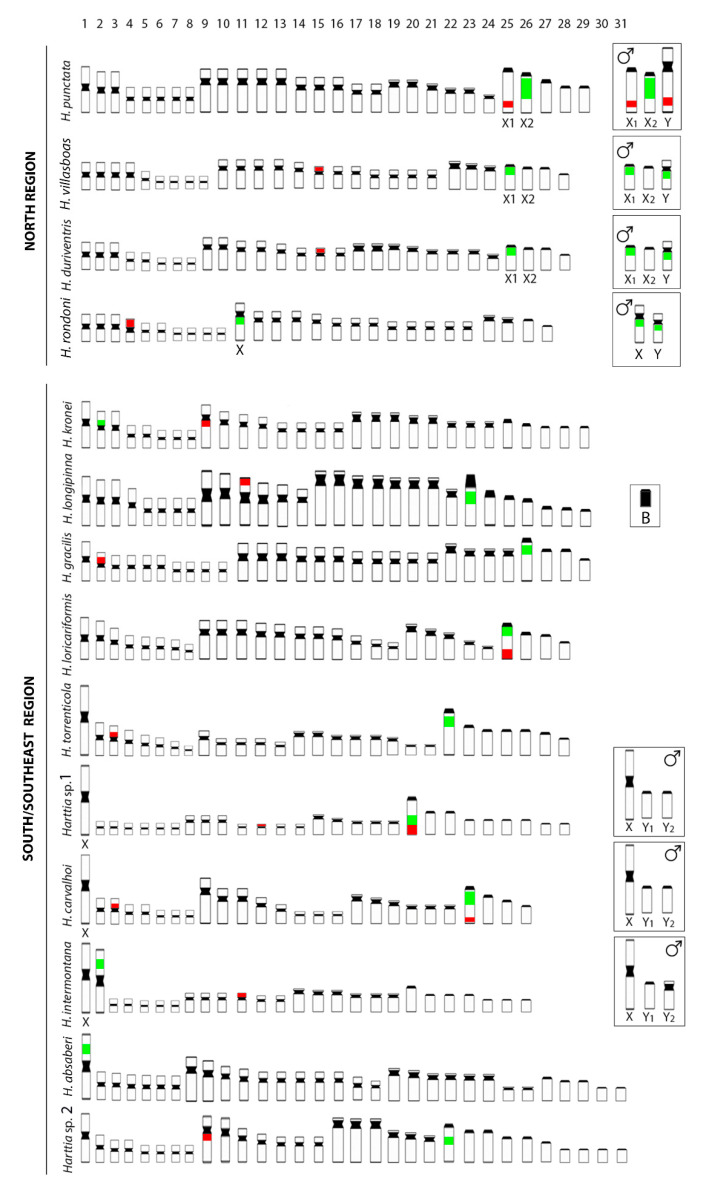
Representative idiograms of *Harttia* species from distinct Brazilian regions based on the distribution of rDNA sequences in their karyotypes, according to the present study, Blanco et al. [8], and Sassi et al. [11] data. The location of the 18S and 5S rDNA sites on the chromosomes are indicated in green and red, respectively. Inserts depict the male sex chromosomes.

The Loricariidae family is an outstanding group to investigate chromosomal breaks and rearrangements that gave rise to extremely diverse karyotypes among its representatives [39,40,41,42]. These fishes are characterized by a sedentary lifestyle, with rare migratory events [43]. Their species occur in small and isolated populations [6] where the fixation of chromosomal rearrangements could occur at higher rates [44,45,46,47]. In fact, the Loricariinae subfamily shows extensive numerical chromosome variation (36 to 74), which is attributed to chromosomal rearrangements, mainly to Robersonian fusions (Rb fusion) and fissions [41,48,49,50]. The *Harttia* genus, in which several cryptic and undescribed species are believed to occur, displays the second-largest chromosomal variation among the Loricariinae (52 to 62, Table 1, Figure 6). In addition, there is also strong evidence for evolutionary breakpoint regions (EBRs) promoting intrachromosomal remodeling, which are still being studied [51].

A putative ancestral karyotype, probably with 2n = 58 chromosomes, is attributed to the *Harttia* lineage, and this same 2n number occurs in its sister group *Farlowella* [52] and in basal species from *Harttia* phylogenetic relationships [7,8]. However, *Harttia* presents different pathways in relation to the 2n diversification, some species keeping 58 chromosomes, some others increasing this chromosome number by centric fissions (i.e., *H. absaberi* and *Harttia* sp. 2 now studied), with others decreasing this number due to Rb fusions (Table 1, Figure 6).

ITS generally reveal chromosomal rearrangements, such as Rb fusions or in tandem fusions [53]. In previous studies, ITS were identified in three *Harttia* species (*H. loricariformis*, 2n = 56; *H. torrenticola*, 2n = 56, and *H. carvalhoi*, 2n = 52♀/53♂), as vestiges of Robertsonian rearrangements [8,9]. It was proposed that fusion events were responsible for originating the largest metacentric pair found in *H. torrenticola* (pair 1) and *H. carvalhoi* (X chromosome), due to the presence of a proximal ITS on their short arms [8]. *Harttia intermontana* and *Harttia* sp. 1 also share a similar large metacentric X chromosome, but no ITS were detected. It is likely that this absence is due to the fact that not all chromosome fusions retain some telomeric DNA repeats at the fusion points [54]. Moreover, the occurrence of different chromosomal rearrangements and modifications of the non-functional telomeric arrays can be also considered [55]. In the last situation, a successive loss and degeneration of the non-functional telomeric repeats that were retained at the fusion sites leads to their gradual shortening, and, consequently, to an insufficient amount to be highlighted by FISH [53,56].

To date, the first largest metacentric pair of *Harttia* is shared by all species that have 2n = 56 chromosomes or a smaller number, except for *H. loricariformis*, and this could be considered as being derived from an Rb fusion chromosome. In *Harttia* sp. 2 the first chromosome pair is also a large metacentric-bearing ITS, however, this chromosome has a small size compared to the chromosome 01 of *H. carvalhoi*, *H. intermontana*, *H. torrenticola*, and *Harttia* sp. 1, thus indicating that additional rearrangements probably played a role on its origin. Noteworthy, *Harttia* sp. 2 presents four bi-armed chromosome pairs bearing ITS at the proximal regions of the long arms. According to the instability genomic proposal, ITS are hotspots for chromosomal breakage [57], and telomeric DNA damage can be irreparable, causing persistent activation in response to DNA damage [58] or remaining as EBRs on the genome [51,59]. This suggests that both ITS and terminal telomeric sequences are naturally prone to breakage, leading to chromosome plasticity [56,60,61]. Here, we propose that *Harttia* sp. 2 increased its chromosome number by centric fissions from an ancestral ITS bearing lineage, which acted as instable sites and promoted double strand breaks (DSBs) triggering further chromosomal rearrangements. This proposal is corroborated by the extensive FN modification among *Harttia* species (Table 1), since only Robertsonian rearrangements keep the FN unchanged throughout the karyotype evolution. It is known that chromosomal rearrangements might play an important role in speciation [47,62]. In this sense, the expressive rearranged karyotypes that are found among *Harttia* species may have acted as significant post-zygotic isolating mechanisms throughout the evolutionary history of this group.

### 4.2. Heterochromatin and rDNA Sites Rearrangements in Harttia Species

The presence of small amounts of heterochromatin is probably an intrinsic characteristic of the *Harttia* species [8]. Indeed, *H. intermontana* and *Harttia* sp. 1 present the same pattern already described for other species of the genus, while in *Harttia* sp. 2, some more prominent pericentromeric bands are colocated with the ITS in the chromosome pairs 1, 9, 16, and 22. The epigenetic regulation of repetitive sequences, such as histone modifications and DNA methylation to form heterochromatin, is proposed to protect ITS from breakages and play important roles in regulation of gene expression [56,63]. In this way, the colocalization of the heterochromatin and ITS may be an expression of an epigenetic property of the *Harttia* sp. 2 genome. In addition, the rDNA loci colocalization with ITS (5S in pair 9 and 18S in pair 22) indicates that these multigene families are also probably associated with chromosomal rearrangements in *Harttia* sp. 2. In the same way, the wide differentiation of the chromosomes carrying the rDNA sequences among *Harttia* species demonstrates that these repetitive sequences may also be closely related to deep chromosomal changes that have occurred within the genus. In fact, in some groups of Loricariidae, the involvement of rDNA pseudogenes colocalized to ITS in chromosomal rearrangements have been demonstrated [40,41,50].

As a whole, three general conditions are found concerning the location of the rDNA genes among *Harttia* species: In the first, a syntenic condition for both 5S and 45S rDNAs occurs, as found in *H. carvalhoi*, *H. loricariformis*, and *Harttia* sp. 1, where the first acrocentric chromosome pair is the carrying one, although with *Harttia* sp. 1 showing a particular syntenic configuration (Figure 3 and Figure 6). Yet, *H. carvalhoi* and *Harttia* sp. 1 present an extra 5S rDNA locus. The second condition includes *H. gracilis*, *H. kronei*, *H. longipinna*, *H. punctata*, *H. villasboas*, *H. duriventris*, *H. torrenticola*, and *Harttia* sp. 2, in which the first acrocentric carries the 18S rDNA site, while the 5S occurs in variable locations of different meta/submetacentric chromosomes (except for *H. rondoni* that has 18S rDNA site in the largest sm). In the third pattern, the 5S locus is found in a submetacentric pair, while the chromosome that carries the 45S rDNA is a large metacentric resulting from a fusion event, as found in *H. intermontana* and *H. absaberi* karyotypes (Figure 3 and Figure 6).

EBRs are DNA clustered regions that are more prone to break and reorganize into genomes, and these specific regions have been described to be re-used during the evolution among related species [64,65,66,67]. According to the model, the evolutionary re-use of DSB regions and multiple locus repositioning among karyotypes corroborate to probable EBR occurrences adjacent to rDNA sites in the *Harttia* lineage, similar to those described in other loricariids, such as *Ancistrus* [40] and *Rineloricaria* [41].

### 4.3. The Rare XX/XY_1_Y_2_ System in Fish Species

Based on an overview of available fish karyotype data [68], only about 5% of the analyzed species possess heteromorphic sex chromosomes, including approximately 47 cases of multiple sex chromosomes [69]. Among them, some different systems, such as ♀X_1_X_1_X_2_X_2_/♂X_1_X_2_Y; ♀XX/♂XY_1_Y_2_; ♀X_1_X_1_X_2_X_2_/♂X_1_Y_1_X_2_Y_2_; ♂ZZ/♀ZW_1_W_2_, and ♂Z_1_Z_1_Z_2_Z_2_/♀Z_1_W_1_Z_2_W_2_, were already identified as scattered on the fish phylogeny and independently evolved in many lineages and, sometimes, even within a same genus or species [70].

In the *Harttia* genus, two multiple sex chromosome systems were previously described, the X_1_X_1_X_2_X_2_/X_1_X_2_Y one in *H. punctata*, *H. duriventris,* and *H. villasboas* and the XX/XY_1_Y_2_ system in *H. carvalhoi* [8,11]. While the first one is well-represented among a variety of fish families [18], the XX/XY_1_Y_2_ system is found in only a few fish species (Table 3). Here, like in *H. carvalhoi* [8], two additional cases were identified in *H. intermontana* and *Harttia* sp. 1.

Multiple XX/XY_1_Y_2_ sex chromosome systems are proposed to have originated by one bi-armed chromosome fission leading to Y_1_ and Y_2_ formation [71,72,73] or by X-autosome fusion forming a large bi-armed X chromosome and subsequent centric fission in the origination of the Y_1_ and Y_2_ chromosomes [74,75,76,77]. In *Harttia* species, the large metacentric 1 observed in *H. torrenticola* is comparable to X chromosome in *H. carvalhoi*, *H. intermontana*, and *Harttia* sp. 2 and was proposed to be originated from an Rb fusion [8].

To date, *Harttia* lineages from the south/southeast Brazilian drainages have no proto-sex or XY chromosomes identified, which would corroborate the proposal of an X-autosome fusion acting in the origin of the XY_1_Y_2_ system. However, the occurrence of *H. torrenticola* (without differentiated sex chromosomes) and *H. carvalhoi* (XY_1_Y_2_) in the same branch of the phylogenetic relationship [7] and the same CGH pattern among *H. carvalhoi*, *H. intermontana*, and *Harttia* sp. 1 concerning sex chromosomes, point to an Rb fusion leading to their large metacentric X-chromosome, as well as to the similar large metacentric pair 1 of *H. torrenticola.*

Although *H. intermontana* and *H. carvalhoi* possess the same 2n and sex chromosome system (XX/XY_1_Y_2_), significant differences occur between the karyotype structure of these two species. The absence of several large submetacentric pairs in *H. intermontana* as well as the occurrence of its large second metacentric pair carrying 18S rDNA cistrons are remarkable. Besides that, the morphology of their Y_2_ chromosome also differs, corresponding to a subtelocentric in *H. intermontana* and to an acrocentric chromosome in *H. carvalhoi*. By comparing the chromosomal morphology and the distribution of the ribosomal sites, it is possible to infer that some additional rearrangements, such as Rb fusion and/or reciprocal translocation, pericentric inversion, and loss or gain of 5S sequences, took place in the chromosome evolution of these species. All data corroborate EBRs occurrence in adjacent regions to rDNA loci and in the pericentromeric region of the largest metacentric pair in the chromosomal diversification of the *Harttia* species inhabiting south and southeast Brazilian drainages.

## 5. Conclusions

Our study provided additional evidence on the evolutionary pathways followed by fish species of the genus *Harttia*, highlighting both shared and specific chromosomal features that have emerged throughout their life story. We were also able to identify two new cases of the rare XX/XY_1_Y_2_ multiple sex chromosomes systems among fishes, displaying a significant particular incidence in the *Harttia* lineages from south/southeast Brazil. The species in this branch, which include the *H. intermontana*, *Harttia* sp. 1, and *Harttia* sp. 2 here studied, experienced different ways of chromosome diversification, such as 2n reduction and increase by Rb fusions and centric fissions, respectively, and the emergence of a XX/XY1Y2 sex chromosome system in different species, in contrast to what occurred with the lineages from north Brazilian regions where the X_1_X_1_X_2_X_2_/X_1_X_2_Y system stands out. The occurrence of deeply reorganized karyotypes in the species here studied are in accordance with EBRs present in the *Harttia* genome, which could be reused for chromosome speciation in this group. As a whole, the present study highlights the importance of cytogenetics as a tool for evolutionary studies and, particularly in the present case, detaching the highly differentiated patterns followed by the *Harttia* lineages from two main Brazilian geographic regions.

## Figures and Tables

**Table 1:** Available cytogenetic data for *Harttia* species.

Species	2n	Karyotype	FN ^1^	References
*Harttia absaberi*	♀♂62	13m + 23sm + 16st + 10a	114	[23]
*Harttia carvalhoi*	52♀, 53♂	16m + 16sm + 12st + 8a ♀ 15m + 16sm + 12st + 10a ♂	96 96	[9]
*Harttia duriventris*	56♀, 55♂	16m + 16sm + 16st + 8a ♀ 17m + 16sm + 16st + 6a ♂	104 104	[11]
*Harttia gracilis*	♀♂58	20m + 22sm + 8st + 8a	108	[8]
*Harttia intermontana*	52♀, 53♂	14m + 12sm + 12st + 14a ♀ 13m + 12sm + 13st + 15a ♂	90 91	Present study
*Harttia kronei*	♀♂58	16m + 16sm + 16st + 10a	106	[8]
*Harttia longipinna*	♀♂58 + 0 − 2 Bs	16m + 12sm + 16st + 14a	102	[24]
*Harttia loricariformis*	♀♂56	16m + 22sm + 10st + 8a	104	[25]
*Harttia punctata*	58♀, 57♂	16m + 20sm + 12st + 10a ♀ 16m + 21sm + 12st + 8a ♂	106 106	[10]
*Harttia rondoni*	♀♂54	20m + 26sm + 4st + 4a	104	[11]
*Harttia torrenticola*	♀♂56	16m + 10sm + 16st + 14a	98	[9]
*Harttia villasboas*	56♀, 55♂	18m + 24sm + 6st + 8a ♀ 19m + 24sm + 6st + 6a ♂	104 104	[11]
*Harttia* sp. 1 (Macacos stream)	56♀, 57♂	14m + 14sm + 10st + 18a ♀ 13m + 14sm + 10st + 20a ♂	94 94	Present study
*Harttia* sp. 2 (Barra Grande river)	♀♂62	16m + 14sm + 12st + 20a	104	Present study

^1^ FN = fundamental number.

**Table 2:** Collection sites, sample sizes (*n*), and sex of the *Harttia* species analyzed.

Species	Locality	*n*
*1.* *Harttia intermontana*	Piranga river, Carandaí, MG (Brazil) (20°59’34.0″ S, 43°43′30.0″ W)	20♀, 13♂
*2.* *Harttia* sp. 1	Macacos stream, Silveira, SP (Brazil) (22°40’43.0″ S, 44°51′25.0″ W)	10♀, 7♂
*3.* *Harttia* sp. 2	Barra Grande river, Prudentópolis, PR (Brazil) (24°58′40.72″ S, 51°7′34.25″ W)	17♀, 11♂

**Table 3:** Multiple XX/XY_1_Y_2_ sex chromosome systems currently found in teleosts.

Species	2n	Mechanism of Origin	Reference
*Bathydraco marri*	38♀, 39♂	Y-chromosome fission	[72]
*Coregonus sardinella*	80♀, 81♂	Y-chromosome fission	[71]
*Schistura* cf. *fasciolata*	50♀, 51♂	Y-chromosome fission	[73]
*Hoplias malabaricus* (karyomorph G)	40♀, 41♂	Tandem fusion X-A	[19,74,78]
*Gymnotus bahianus*	36♀, 37♂	Tandem fusion X-A	[76]
*Ancistrus dubius*	38♀, 39♂	X-A tandem fusion and further neo-Y chromosome fission	[75,77]
*Harttia carvalhoi*	52♀, 53♂	Y-chromosome fission	[8,9,79,80]
*Harttia intermontana*	52♀, 53♂	X-A tandem fusion and further neo-Y chromosome fission	Present study
*Harttia* sp. 1	56♀, 57♂	X-A tandem fusion and further neo-Y chromosome fission	Present study
